# Vitamin D and Parkinson’s Disease

**DOI:** 10.3390/nu14061220

**Published:** 2022-03-14

**Authors:** Antonia Pignolo, Sergio Mastrilli, Chiara Davì, Valentina Arnao, Paolo Aridon, Felipe Augusto dos Santos Mendes, Cesare Gagliardo, Marco D’Amelio

**Affiliations:** 1 Department of Biomedicine, Neurosciences and Advanced Diagnostics, University of Palermo, 90127 Palermo, Italy; antonia.pignolo@unipa.it (A.P.); sergiomastro@gmail.com (S.M.); chiaradavi092@gmail.com (C.D.); paolo.aridon@unipa.it (P.A.); cesare.gagliardo@unipa.it (C.G.); 2 UO Neurologia e Stroke Unit, Azienda di Rilievo Nazionale ad Alta Specializzazione, Ospedali Civico Di Cristina Benfratelli, 90134 Palermo, Italy; arnao.valentina@gmail.com; 3 Graduate Program in Rehabilitation Sciences, University of Brasília, Brasília 72220-275, Brazil; mendesf.fm@gmail.com

**Keywords:** Parkinson’s disease, neuroprotection, neurodegeneration, vitamin D, vitamin D receptor, 1,25-dihydroxyvitamin D, motor symptoms, balance, cognition, disease progression

## Abstract

Vitamin D is a fat-soluble secosteroid, traditionally considered a key regulator of bone metabolism, calcium and phosphorous homeostasis. Its action is made possible through the binding to the vitamin D receptor (VDR), after which it directly and indirectly modulates the expression of thousands of genes. Vitamin D is important for brain development, mature brain activity and associated with many neurological diseases, including Parkinson’s disease (PD). High frequency of vitamin D deficiency in patients with Parkinson’s disease compared to control population was noted nearly twenty years ago. This finding is of interest given vitamin D’s neuroprotective effect, exerted by the action of neurotrophic factors, regulation of nerve growth or through protection against cytotoxicity. Vitamin D deficiency seems to be related to disease severity and disease progression, evaluated by Unified Parkinson’s Disease Rating Scale (UPDRS) and Hoehn and Yahr (H&Y) scale, but not with age of PD onset and duration of disease. Additionally, fall risk has been associated with lower vitamin D levels in PD. However, while the association between vitamin D and motor-symptoms seems to be possible, results of studies investigating the association with non-motor symptoms are conflicting. In addition, very little evidence exists regarding the possibility to use vitamin D supplementation to reduce clinical manifestations and disability in patients with PD. However, considering the positive balance between potential benefits against its limited risks, vitamin D supplementation for PD patients will probably be considered in the near future, if further confirmed in clinical studies.

## 1. Introduction

As consequence of the growth of global population and improvement of the average lifespan, prevalence of neurological disorders is increasing. The scientific community is focused on the research of treatment and prevention of brain aging. Many mechanisms are involved in degeneration: inflammation, oxidative stress, mitochondrial dysfunction, lysosomal depletion, metal dysregulation, impaired RNA homeostasis, misfolding and aggregation of specific proteins, such alpha-synuclein, amyloid *β* (A*β*), hyperphosphorylated tau [1]. Among others, lower serum concentrations of vitamin D (or 25-hydroxyvitamin D) seems to be associated with psychiatric disorders such as depression, bipolar disorder and schizophrenia, as well as neurological disorders, including neurodegenerative disorders such as dementia and Parkinson’s disease (PD) [2]. Consequently, it has been postulated that maintaining adequate vitamin D serum concentration might avoid disease onset and possibly improve clinical outcomes [2]. Here, we review the role of vitamin D as protective factor against neurodegeneration, its changes in serum concentration during disease progression and its hypothetical therapeutic use.

## 2. Vitamin D

Vitamin D refers to a group of fat soluble secosteroids, supplied with the diet or produced by skin exposure to ultraviolet light. Two forms of vitamin D exist, vitamin D2, (ergocalciferol) and vitamin D3 (cholecalciferol). Vitamin D can also be synthesized by humans in skin, during sunlight exposure, by the effect of ultraviolet B (UVB) realizing the conversion from the steroid precursor, 7-dehydrocholesterol in activated vitamin D3 [3,4]. Vitamin D is activated by a double hydroxylation, occurring first in the liver, by 25-hydroxylase, into 25-hydroxyvitamin D (25(OH)D) and then in the kidney, by the action of 1-α-hydroxylase resulting in an active form of the vitamin, the 1,25-dihydroxyvitamin D2 (1,25(OH)_2_D_2_) or the D3 (1,25(OH)_2_D_3_) [3,5].

Biologically not active, 25(OH)D concentration can be detected in humans with normal serum concentration between 50–250 nmol/L [6]. There is consensus that, as it is capable of reflecting the contribution that both diet and skin synthesis provide, the serum or plasma level of 25 (OH) D should be used to assess vitamin D status [7].

Globally, among health agencies and scientific organizations, there is no agreement on the cut-off values that define the normal threshold of serum 25(OH)D concentration, and this can range from >25 to >50 nmol/L [7]. Vitamin D sufficiency has been variously defined, but there is general agreement that deficiency corresponds to 25(OH)D levels less than 25–30 nmol/L [7].

Up to 95% of 25(OH)D binds to vitamin D-binding protein (VDBP), 10% circulates in association with albumin, while only 1% is free. Vitamin D regulates bone health, but it has significant functions in other systems such as the central nervous system (CNS) [8]. Brain contains the enzyme 1a-hydroxylase, producing the active form of vitamin D, and vitamin D receptors (VDR) [8]. In fact, circulating 25-hydroxyvitamin D (25(OH)D) is able to penetrate the blood–brain barrier to be hydroxylated in neuronal and glial cells into the active form 1,25OH_2_D [9]. 

In the CNS, vitamin D can activate different pathways, genomic and non-genomic [10,11]. Indeed, in CNS there are two different types of vitamin D receptors: a nuclear receptor (VDR) and a membrane-associated rapid response steroid binding receptor (MARRS). VDR is a member of the nuclear receptor family of transcription factors a steroid/thyroid hormone superfamily of transcription regulation factors that, when complexed with calcitriol, binds vitamin D to specific genomic sequences, inducing gene transcription (genomic pathway) [12]. Vitamin D binds also a membranous receptor, the surface receptor MARRS, with different effects in calcium and phosphates homeostasis, molecular chaperoning, immunomodulation and activation of the protein kinase C pathway, cAM pathway, as well as the MAP kinase pathway (non-genomic pathway). However, 1,25D3-MARRS receptor also has been found to have genomic effect, binding DNA and regulating gene transcription [10,13]. 

Through its receptors, the vitamin D carries out many effects. 1,25(OH)_2_D_3_ can upregulate the transcription of the proto-oncogene tyrosine-protein kinase receptor Ret (C-Ret) and GDNF genes, both involved in antioxidant and neuroprotective regulation [14]. 1,25(OH)_2_D_3_ also increases expression of Nurr1 and p57kip2, cooperating and regulating the differentiation and maturation of DA neurons [15,16].

Though the main function of vitamin D is related to bone health, its role in brain development has been widely described, as well as in neuroprotection of adult brain [17,18,19,20,21]. Vitamin D metabolites could pass through blood–brain barrier and the presence of 1,25(OH)_2_D_3_ in the cerebrospinal fluid (CFS) suggests the existence of a catabolic pathway in the CNS [22]. Not only is 1α-hydroxylase expressed in the brain (especially cerebellum, cerebral cortex and substantia nigra) [23], but it was also observed that different neuronal population could inactivate 1,25OH_2_D_3_ to 25OHD_3_ [24]. The presence of a high concentration of VDR and 1-α-hydroxylase in the substantia nigra (SN) provides evidence of a possible relationship between PD and vitamin D [8].

## 3. Vitamin D and Parkinson Disease

Parkinson Disease (PD), the second most common neurogenerative disorder, is characterized by the motor symptomatologic triad, tremor, rigidity, and bradykinesia, usually with initial asymmetric involvement. Clinical manifestation includes other motor features such freezing of the gait (FOG), postural instability, camptocormia and Pisa-syndrome. PD is characterized also by non-motor symptoms (NMSs) that might precede motor symptoms by more than a decade (autonomic impairment, orthostatic hypotension, sleep disorders, olfactory impairment, sialorrhea, dysphagia, fatigue, pain, cognitive and neuropsychiatric disturbances) [25,26]. In advanced stage of Parkinson’s, disease treatment-resistant motor and non-motor symptoms results prominent, and after 17 years of disease duration, about 80% of PD patients have freezing of gait (FOG) and falls [26].

Neuropathology of PD is characterized by the progressive death of dopaminergic (DA) neurons in the substantia nigra pars compacta (SNpc) of the midbrain and by the development of neuronal Lewy Bodies. These consist of α-synuclein protein aggregates, leading to neuronal death with the consequent reduced ability to synthesize dopamine [27]. Several etiopathogenetic pathways are implicated in neurodegeneration, such as excitotoxicity, apoptosis, oxidative stress, mitochondrial dysfunction, inflammation, and others [28].

The ethology of neurodegeneration in PD is not yet clear, but interactions of both genetic and environmental factors such as rural living, brain injury, pesticide exposure, alcohol consumption, smoking, etc. are very likely involved [26]. 

Among the main risk factors in PD such as pesticides, drugs, smoking, alcohol, traumatic brain injuries, vitamin D deficiency may be another [29]. Other risk factors seem to be inversely related to Parkinson disease, such as tumors, smoke and estrogenic hormones [30,31].

Since oxidative stress represents one of the etiopathogenetic pathways of PD, substances with anti-inflammatory and antioxidant action could be used to limit this mechanism. Vitamin D has an antioxidant function in the brain and its deficiency could be implicated in the onset of PD [32].

To date, many symptomatic medications are available to reduce severity of PD symptoms, and no disease-modifying therapies has been proven clearly effective [33]. Similarly, considering its neuroprotective function, Vitamin D might be used as adjunctive treatment in PD patient to improve symptoms and possibly the disease’s course [34]. 

In this review, we will describe the most significant and more recent findings on vitamin D and PD. We will examine its possible neuroprotective action, its association with some clinical characteristics of the disease and, finally, a hypothetical role of vitamin D as an adjunctive treatment in PD.

## 4. Neuroprotective Effect of Vitamin D in Parkinson Disease

**Figure 1:**
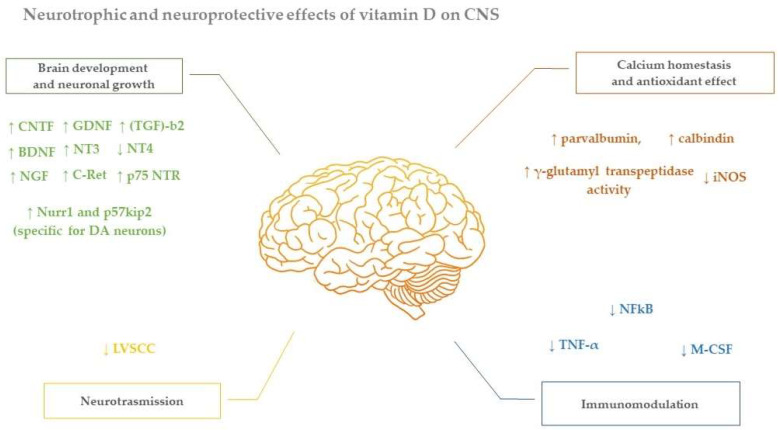
Principal neurotrophic and neuroprotective effects of Vitamin D on CNS. nerve growth factor (NGF), glial cell-derived neurotrophic factor (GDNF), transforming growth factor (TGF)-b2, ciliary neurotrophic factor (CNTF), neurotrophin 4 (NT4), neurotrophin 3 (NT3), brain-derived neurotrophic factor (BDNF), proto-oncogene tyrosine-protein kinase receptor Ret (C-Ret), p75 neurotrophin receptors (p75 NTR), L-type voltage sensitive Ca2+ channel (LVSCC), nuclear factor kappa-light-chain-enhancer of activated B cells (NFkB), macrophage colony-stimulating factor (M-CSF), tumor necrosis factor α (TNF-α), inducible nitric oxide synthase (iNOS), increased activity (↑), decreased activity(↓).

The role of vitamin D in Parkinson’s disease has been widely studied. Lower 25(OH)D levels might be responsible for dopaminergic neuronal death contributing to PD development, due to the lack of its protective function [35]. The specific action of vitamin D protecting against PD is not clear. However, many mechanisms have been correlated with a neuroprotective effect against excitotoxic insults, as showed in Figure 1: 1,25(OH)_2_D_3_ stimulates the release of neurotrophin and the synthesis of Ca^2+^-binding proteins such as parvalbumin, it inhibits the synthesis of inducible nitric oxide synthase (iNOS), macrophage colony-stimulating factor (M-CSF) and tumor necrosis factor α (TNF-α), and it induces downregulation of LVSCC and upregulation of γ-glutamyl transpeptidase activity [36,37,38,39,40,41]. Additionally, a lower concentration of vitamin D correlates with high levels of C-reactive protein (CRP), a marker of inflammation [42]. Overall vitamin D role appears fundamental in the prevention of brain aging, considering also its function in the production of growth factors, including nerve growth factor (NGF), ciliary neurotrophic factor (CNTF), glial cell-derived neurotrophic factor (GDNF), glial cell-line-derived neurotrophic factor (GDNF), brain-derived neurotrophic factor (BDNF), and neurotrophin 3 (NT3) [9,43,44,45]. 

In fact, vitamin D induces an increase in circulating neutrophins such as NGF, GDNF, BDNF, NT3, CNTF, low-affinity p75 neurotrophin NT receptor (p75 NTR), and transforming growth factor (TGF)-b2, and it induces the downregulation of neurotrophin 4 (NT4) [8,17,45,46,47,48]. 

Vitamin D contributes in intraneuronal calcium (Ca^2+^) homoeostasis and cytosolic Ca^2+^ glial concentration, acting on the regulation of L-type voltage sensitive Ca^2+^ channel (LVSCC), modifying neuronal function and upregulating the synthesis of parvalbumin and calbindin. High concentrations of Ca^2+^ have toxic effects causing an elevation of ROS levels and mitochondrial dysfunction and inducing neuronal cell death. Vitamin D leads to decrease in excitotoxicity injury triggered by cytoplasmic Ca^2+^, especially when there is a sudden increase in calcium level [36]. Vitamin D has also antioxidant effects, reducing the formation of free radicals and production of reactive oxygen species (ROS), due to the capacity to reduce the synthesis of nitric oxide synthase, reducing activity of NFkB (nuclear factor kappa-light-chain-enhancer of activated B cells), and enhancing the activity of the gamma glutamyl transpeptidase [49]. 

In addition, vitamin D is a renin-angiotensin system regulator (RAS), and its altered function could lead to sympathetic dysfunction [50].

Considering all these significant actions, a neuroprotective effect of vitamin D is likely and consequently might reduce progression towards neurodegenerative process.

As a consequence, inadequate vitamin D status could lead to a loss of dopaminergic neurons in the brain, and therefore could contribute to development of PD [51]. 

Reduced concentration of vitamin D in PD patients, compared to those of sex- and age-matched healthy controls, has been described [52,53]. 

Many studies investigated the influence of a reduced 25-hydroxyvitamin D level, hypothesizing that a low concentration of vitamin D may be a risk factor, and defined an increasing risk of PD for vitamin D deficiency (<50 nmol/L) compared to insufficiency (<75 nmol/L) [51,54,55]. In a cross-sectional study it was demonstrated that serum 25(OH)D concentrations are decreased in PD patients compared to patients with Alzheimer’s disease, but it also showed a decreased levels compared to age- and sex-matched healthy controls [56]. These results could be explained by the longer clinical history and higher immobility in PD patients, compared to AD patients, causing a reduction to sunlight exposure and consequently lack in skin synthesis [57]. 

Though reduced mobility and therefore sunlight exposure in patients with PD are plausible explanations for reduced 25(OH)D levels, concentration of 25(OH)D are significantly lower in PD patients with adequate sunlight exposure, compared to healthy controls [58]. Ding et al. detected both lower 25(OH)D_3_ and lower total 25(OH)D serum concentrations in PD patients compared with controls [55]. In a post hoc analysis, it has been shown a higher incidence of PD in subjects with the lowest quartile of 25(OH)D serum concentration, compared to the highest quartile of 25(OH)D concentration [51]. 

Further studies analyzed the association of sunlight exposure (>15 min/week) with decreased risk of PD [59]. It was hypothesized that persistent insufficient levels of vitamin D could play a role in the pathogenesis in neurodegenerative disease, including PD [58,60]. In a case–control study vitamin D dispensation, outdoor activity and consequent sunlight exposure were inversely associated with PD [61]. 

However, for many authors, there is not sufficient evidence to confirm a function of vitamin D in PD pathogenesis [62]. Shrestha et al., in a prospective study, found no significant association between serum 25(OH)D and PD risk [63]. 

Considering the possible role of vitamin D in neurodegeneration, the attention of many authors has been turned into the role of vitamin D receptor (VDR). VDR is expressed in the CNS neurons, astrocytes and oligodendrocytes, and above all in substantia nigra, cortex, subcortex, hippocampus, hypothalamus, thalamus, and vessel walls [64,65]. The presence of VDR and 1-alpha-hydroxylase, the enzyme that converts 25(OH)D to its active form 1,25-dihydroxyvitamin D (1,25(OH)D), in substantia nigra underlines the role of vitamin D in PD suggesting that vitamin D hydroxylation and activation is also completed in central nervous system (CNS), and therefore the deficit of vitamin D concentration causes dopaminergic neurons death [62,66]. 

Further confirmation of the role of vitamin D in PD etiopathogenesis is supported by motor impairment in VDR knockout mice, and genotypes were recognized in the VDR gene that are linked with some characteristics of PD [67,68]. 

Many studies confirmed the presence of VDR in animal and human brains and that vitamin D administration increased dopamine (DA) production. This would be due to the increased expression of *tyrosine hydroxylase* (TH) (the rate-limiting synthetic enzyme for dopamine) with the consequent increase in neuronal survival DA neurons, both in vitro and in vivo [8,69,70,71,72,73]. 

Vitamin D administration reduces dopaminergic toxicity of 6-hydroxy dopamine [74], and the lack of vitamin D receptor (VDR) induced a significantly impaired motor function in mice [67,75]. Several genetic association studies, supporting the possible role of vitamin D in PD risk, searched for the connection between VDR single nucleotide polysmorphisms (SNPs) and PD [56,76,77,78]. A genome-wide association study (GWAS) demonstrated the association of VDR polymorphisms with both risk and age at onset of PD [68]. 

Therefore, as shown in some studies [35,76,77,79], response to vitamin D administration in PD patients depends upon the genotype of VDR.

VDR CC allele (FokI) is associated with PD, and subjects with FokI CC demonstrated no significant response to vitamin D administration with respect to placebo compared with other VDR genotypes [76,77]. Specifically, subjects with FokI TT allele had a significant response to vitamin D administration compared to individuals with VDR FokI CT, who had a moderate response [79]. FokI CC was also associated with milder form of PD [35]. 

Genetic factors might be a cause of vitamin D deficiency, and the association of PD with single nucleotide polymorphisms (SNPs) in the VDR and vitamin D binding protein (VDBP/GC) have been investigated in PD, such as a possible cerebrospinal fluid (CSF) biomarker in PD [56,76,77,78,80]. 

Further support to the hypothesis of a neuroprotective role of vitamin D comes from numerous studies. Among these two cross-sectional studies supports the hypothesis that vitamin D insufficiency is associated with an increased risk of Parkinson’s disease [56,81], as well as an association between high serum 25(OH)D levels and a reduced risk of PD has been observed in a longitudinal prospective study by Knekt P et al. [51]. 

Another supporting factor is that outdoor work seems to be related to a lower risk of developing PD, suggesting that greater sun exposure and the consequent increased vitamin D synthesis in skin represent a protective factor [82,83]. 

## 5. Vitamin D in Relation with Parkinson Disease Symptoms and Disease Progression

While the role of Vitamin D as a protective factor in PD is promising, though still controversial, its role in PD symptoms progression is much more complex.

In an animals model, Kaluef et al. showed that genetic ablation of VDR in mice is associated with specific impairments in motor performance, and this, according to their explanation, could possibly be attributed to the localization of VDR in the brain and spinal cord and suggests that vitamin D plays a crucial role in the functioning of the motor system [75].

Meamar et al. conducted a cross-sectional study to investigate the relationships between serum 25(OH)D_3_ levels and serum UA concentrations as well as their interaction with severity of PD. In their population of Iranian PD patients, a negative significant association between serum 25(OH)D_3_ and UPDRS score was found, indicating that higher serum levels of 25(OH)D_3_ was associated with lower PD severity; this finding was restricted to patients older than 62 years [82].

Therefore, the important role of Vitamin D in humans was demonstrated by the negative correlation between serum 25(OH)D_3_ levels and PD severity, determined by UPDRSIII score, conversely the 25(OH)D concentrations seems do not correlate with freezing, postural instability and abnormal postures, all signs of PD disease progression [53,82].

Other authors demonstrated an inverse association between higher UPDRS scores and Hoehn and Yahr (HY) staging, and low levels of 25(OH)D_3_ and total 25(OH)D levels, concluding that decreased concentration of vitamin D are related to increased disease severity but not with disease duration and age of PD patients [55,83,84]. 

These results were confirmed in the DATATOP study, asserting that 25(OH)D levels did not decrease during progression of PD [85].

Suzuki et al. described a slower PD progression, evaluated by means of the H&Y scale, in the activities of daily living by UPDRS Part II, and the quality of life by Parkinson’s Disease Questionnaire (PDQ39), in the group of PD patients receiving vitamin D reintegration, compared to PD patients who received a placebo [34]. 

In a genome-wide association (GWA) study, Gezen-Ak et al. indicated a relationship between low serum levels of 25(OH)D and PD, identifying several polymorphisms in vitamin D receptor (VDR) genes that correlate with different degrees of severity in Parkinson’s in relation to serum levels of 25(OH)D [86].

Vitamin D deficiency might have an impact in progression of PD and its clinical motor and non-motor manifestations.

Sleeman et al. concluded that the worst PD progression, referring to motor impairment severity evaluated by UPDRS part III, was related to lower serum 25(OH)D to baseline [83].

Moghaddasi et al. found that low 25(OH)D levels were associated with severe postural instability, freezing gait, and postural abnormalities [53].

Among motor symptoms, Peterson et al. investigated the relationship between vitamin D levels and balance control. Considering that postural instability represents a common symptom in PD, and that in heathy subjects the automatic postural responses are symmetric while PD patient have asymmetrical reaction, the authors found a correlation between lower vitamin D concentrations and the rate of asymmetrical postural responses [87]. Automatic postural response impairment might be caused by decreased muscular strength and alteration in latency of postural responses, involving cortex, spinal cord, brainstem, cerebellum and basal ganglia, and VDR is localized in all these structures [88,89].

Some authors observed an increased risk of fracture in PD patients than general population [90]. This could be dependent on a higher risk of falls, motor impairment, balance difficulties, postural instability and by lower levels of calcium and of vitamin D compared to healthy controls [83,91], possibly explained by less sunlight exposure, immobility and insufficient intake of calcium and vitamin D with the diet [59].

Among non-motor symptoms, depression and cognitive impairment seem to be influenced by 25(OH)D_3_. Better scores in neuropsychiatric testing, especially verbal fluency and verbal memory, are associated with higher 25(OH)D_3_ serum levels [92], and 25(OH)D concentration was correlated with depression and anxiety scores [93]. Gatto et al. assessed the role of VDR polymorphisms in cognitive decline in patients with PD and especially in subject with FokI polymorphism [94]. 

Fewer data are available on other non-motor symptoms. Lower levels of 25(OH)D correlated with increased sleepiness, evaluated by Epworth Sleep Scale (ESS) [95] while 25(OH)D_3_ levels are correlated with the severity of olfactory dysfunction in PD patients [96].

Orthostatic hypotension (OH) was inversely associated with decreased serum concentration of 25(OH)D and 1,25(OH)_2_D [97] and serum 25(OH)D_3_ levels can also compromise gastric dysmotility, inducing increased slowness gastric emptying time [98].

To date, very limited evidence exists on the association between non-motor symptoms and vitamin D deficiency.

## 6. Vitamin D Supplementation in Parkinson Disease

Many attempts have been made in order to address a crucial unmet demand in the management of Parkinson’s disease: the discovery of a drug potentially able to slow down, stop or reverse the process of neurodegeneration.

Considering that 1,25(OH)2D3 passes through the blood–brain barrier, hypothesizing that its systemic administration could reduce neuronal injury, some studies investigated the use of Vitamin D as a treatment to reduce some PD symptoms.

We already argued that the stimulation of neurotrophin production by 1,25(OH)_2_D_3_ improves cell survival and reduces toxicity mediated by reactive oxygen species. In a study conducted in rats, 1,25-dihydroxyvitamin D (D3) pretreatment protected against 6-OHDA damage of the substantia nigra (SN) reducing hypokinesia [72]. Treatment with 1,25(OH)_2_D_3_ seems to increase DA release in striatum, and it could be mediated by enhanced GDNF and glutathione levels in rats’ central nervous system, and specifically in mesencephalic dopaminergic neurons [71,99,100].

As previously discussed, 1,25(OH)_2_D_3_ also plays a role in limiting inflammation, and some studies observed how in 1-methyl-4- phenyl-1,2,3,6-tetrahydropyridine (MPTP)-induced preclinical animal model of PD, orally administered vitamin D reduced the immunoreaction of glial fibrillary acidic protein (GFAP) in striatum and SNpc, suggesting that vitamin D might protect against glia-mediated inflammation and nigrostriatal neurodegeneration [69].

Among studies conducted in humans, Suzuki et al. evaluated, in a randomized placebo-controlled clinical trial, the relationship between 1200 IU/day of vitamin D administration and disease progression for two years follow-up. Patients with Parkinson’s disease receiving placebo had a worse neurological outcome, determined by Hoehn and Yahr scale (H&Y) and Unified Parkinson’s Disease Rating Scale (UPDRS), and worse quality of life evaluated by PDQ-39 compared to patients who received vitamin D supplements [34].

Luthra et al. conducted a cohort study in early PD patients followed for three years and divided into three groups: supplement multivitamin (MVI), vitamin D administration ≥400 IU/day, and vitamin D + MVI. The authors did not observe differences in disease progression within the three groups [101].

## 7. Conclusions

In this review, we summarized the current literature concerning the possible role of vitamin D in numerous physiological functions, from the modulation of the immunological response to the regulation of brain development and aging (Table 1). We discussed how serum levels of 25(OH)D are possibly related to PD symptoms and clinical progression, and about its possible use in order to improve clinical manifestation in patients with PD.

In summary, as low serum 25(OH)D levels might be correlated with an increased risk of developing PD, higher 25(OH)D levels seems to be associated with better motor symptoms, especially with improved balance control. It is not yet clear if vitamin D is related to the severity of symptoms of PD and with clinical progression; therefore, its role as disease progression biomarker for PD is not yet clear. Further studies are needed to establish the role of vitamin D etiology of PD, and its relationship with motor and non-motor symptoms, quality of life and progression of disease. 

It is not yet proven if vitamin D reintegration could be an appropriate support to pharmacological and rehabilitative therapy in PD patients. However, though insufficient evidence is available to introduce vitamin D as supportive therapy in PD patients, considering its limited risks, we are confident enough to insinuate, as a dietary intervention, that vitamin D supplementation would act at three different levels: (1) improve public health considering its possible role in brain development and its influence in pathogenesis of many neurological disorders, including PD; (2) slowing down the worsening of some PD symptoms; (3) finally, considering the increased risk of falls during disease progression, reduce the risk of fracture in PD patients.

## Figures and Tables

**Table 1:** Main conclusion of selected studies related to vitamin D and Parkinson’s Disease.

Study Type	Authors	Year	Main Conclusion
SNP	Butler et al. [68]	2011	VDR as a potential susceptibility gene and support an essential role of vitamin D in PD
	Gatto et al. [78]	2015	VDR polymorphisms may modulate risk of PD in a population highly exposed to UVR throughout lifetime
	Cui et al. [66]	2015	VDR is present in the nucleus of tyrosine hydroxylase (TH)-positive neurons in both human and rat substantia nigra
	Fu et al. [102]	2019	No vitamin D3 was detected in metabolites in the prefrontal cortex, middle frontal cortex, middle temporal cortex, cerebellum, corpus callosum, medulla, and pons of this single human brain
post-mortem	Eyles et al. [8]	2005	1a-OHase and VDR are widespread distributed in human brain
	Shirazi et al. [45]	2015	1,25OH2D3. This vitamin significantly enhanced proliferation of NSCs, and enhanced their differentiation into neurons and oligodendrocytes, but not astrocytes
in vitro	Puchacz et al. [73]	2013	1,25OH2D3 regulates catecholamine production in adrenal chromaffin cells providing response and adaptation to stress
	Cui et al. [20]	2007	DVD deficiency has been shown to alter brain structure and function
	Shinpo et al. [100]	2000	1,25OH2D3 increases the intracellular glutathione determining ROS suppression with antioxidative function and defending mesencephalic dopaminergic neurons against BSO/MPP(+)-induced toxicity
	Garcion et al. [99]	1999	1,25-D3 play a fundamental role in astrocyte detoxification pathways
	Musiol et al. [44]	1997	1,25OH2D3 treatment increased the NGF concentration
	Garcion et al. [39]	1997	1,25D3 has an inhibitory effect on iNOS expression and could be synthesized by macrophages or microglia controlling CNS-specific immune responses.
	Furman et al. [40]	1996	1,25OH2D3 play a role in regulation on CNS immune response, by modification of astrocytes response to an inflammatory stimulus
	Garcion et al. [41]	1996	1,25D3 could be an effector controlling detoxification processes in the brain.
	Naveilhan et al. [9]	1996	1,25OH2D3 is a potent inducer of GDNF expression
	Neveu et al. [43]	1994	Activated brain macrophages may be committed to synthesize 1,25OHD, in vitro.
	Naveilhan et al. [103]	1993	vitamin D3 metabolites are involved in brain function
	De Viragh et al. [38]	1989	Vitamin D influences the concentration of calcium-binding-proteins in the periphery and brain
	Moghaddasi et al. [53]	2013	Non-PD patients were detected lower 25OHD level and it was significantly associated with FOG, postural instability and abnormal postures
in vivo	Calvello et al. [69]	2017	Vitamin D exhibits substantial neuroprotective effects in this PD animal model, by attenuating pro-inflammatory and up-regulating anti-inflammatory processes
	Cass et al. [70]	2014	Calcitriol can upregulate GDNF and dopaminergic release in striatum, increasing DA levels in the substantia nigra
	Cass et al. [71]	2012	In animals treated with 6-OHDA followed by calcitriol there was significantly greater potassium and amphetamine evoked overflow of DA from the lesioned striatum compared to that from the control animals
	Cui et al. [16]	2010	the DVD-deficient embryos had a significant reduction in factors crucial in specifying dopaminergic phenotype, such as Nurr1 and p57Kip2
	Smith et al. [74]	2006	Long-treatment with calcitriol can provide partial protection for dopaminergic neurons against the effects of 6-OHDA
	Burne et al. [67]	2005	VDR mice knockout have motor impairments but seemingly no compromission in cognition
	Kalueff et al. [75]	2004	VDR genetic ablation produces severe motor impairment
	Eyles et al. [18]	2003	Rats born to vitamin D3-deficient mothers had alterations in the brain at birth: lateral ventricles were enlarged, the cortex was thinner
	Wang et al. [72]	2001	D3 pretreatment reduces the hypokinesia and DA neuronal toxicity induced by 6-OHDA
	Prüfer et al. [104]	1999	The widespread distribution of vitamin D3 receptor suggests multiple functions of 1,25OHD3 in the CNS.
	Fahmy et al. [52]	2020	Serum 25OHD3 was lower in PD patients and was negatively correlated with age and age at onset of disease, but not with disease duration and PD severity. Serum 25OHD3 was not found to be predictor for severity of PD
case–control	Zhang et al. [93]	2019	Vitamin D levels significantly correlated with falls and some non-motor symptoms
	Kim et al. [96]	2018	The serum 25OHD3 level was independently associated with odor identification score in patients with PD
	Alfieri et al. [42]	2017	25OHD levels were negatively correlated with mRS after three-month follow-up
	Sleeman et al. [83]	2017	PD patients have significantly lower serum 25OHD concentrations than controls, which may have implications in terms of bone health and fracture risk
	Kwon et al. [98]	2016	Vitamin D status may play a role in the pathogenesis of delayed gastric emptying in drug-naive PD.
	Wang et al. [54]	2015	Association between vitamin D levels and PD is not simply due to lack of sunlight exposure PD patients with impaired mobility
	Jang et al. [97]	2015	Low vitamin D status is associated with OH in patients with PD
	Zhu et al. [61]	2014	Outdoor activity and total vitamin D intake were inversely associated with PD
	Ding et al. [55]	2013	Lower levels of 25OHD3 are correlated with higher total UPDRS scores at baseline and during follow-up
	Török et al. [76]	2013	The frequency of FokI C allele was significantly higher in PD patients than in controls, suggesting that this polymorphism may have a role in the development of PD in these patients
	Peterson et al. [92]	2013	Vitamin D plays a role in balance in PD
	Han et al. [77]	2012	VDR FokI T/C polymorphism is related to PD and it may change genetic susceptibility to sporadic PD
	Suzuki et al. [35]	2012	Higher 25OHD levels and vitamin D receptor FokICC genotype may be independently associated with milder forms of PD
	Abou-Raya et al. [59]	2009	PD is associated with increased risk of falls, fractures and osteoporosis
	Di Monaco et al. [90]	2006	BMD expressed as a T score did not differ significantly between PD patients and controls
	Kim et al. [81]	2005	association between PD and a VDRG BsmI polymorphism, which might be involved in the pathogenesis of PD
	Meamar et al. [82]	2015	Negative correlation between interaction of serum vitamin D3 and UA with severity of PD
cross sectional	Luthra et al. [101]	2018	Vitamin D administration does not influence disease progression in PD patients
cohort	Gezen et al. [86]	2017	PD patients with slower progression had significantly higher levels of serum 25OHD
	Gatto et al. [94]	2016	*Fok*l, a functional VDR polymorphism, as being associated with cognitive decline in PD.
	Shrestha et al. [63]	2016	Vitamin D may reduce the risk of PD
	Peterson et al. [87]	2013	Higher plasma vitamin D is associated with better cognition and better mood in this sample of PD patients without dementia
	Evatt et al. [85]	2011	Vitamin D concentrations did not decline during progression of PD
	Knekt et al. [51]	2010	higher serum vitamin D concentrations showed a reduced risk of Parkinson disease
	Evatt et al. [56]	2008	Higher prevalence of hypovitaminosis in PD respect both healthy controls and patients with AD
	Van de Bos et al. [58]	2013	More than half of the patients with early stage PD had an abnormal BMD. Vit. D concentrations were reduced in PD underscoring the importance of proactive screening for bone loss and vitamin D deficiency, even in early stages of PD
RCT	Suzuki et al. [34]	2013	Vitamin D3 supplementation may stabilize PD for a short period in patients with FokI TT or CT genotypes.

Uric acid (UA); central nervous system (CNS); dihydroxyvitamin (OH); modified Rankin Scale (mRS); neural stem cell (NSC); Uric acid (UA); The vitamin D receptor (VDR), freezing of the gate (FOG); developmental vitamin D (DVD); dopamine (DA); L-buthionine sulfoximine (BSO); 1-methyl-4-phenylpyridium ions (MPP(+)); orthostatic hypotension (OH); bone mineral density (BMD); Unified Parkinson’s Disease Rating Scale (UPDRS); vitamin D receptor gene (VDRG); ultraviolet radiation (UVR); bone mineral density (BMD).

## Data Availability

Data sharing is not applicable to this article.
